# Curettage combined with bone cavity opening reduces recurrence of the mandibular conventional ameloblastoma and effectively preserves the mandible: a retrospective study

**DOI:** 10.1186/s12903-023-03660-1

**Published:** 2023-11-20

**Authors:** Yuan Zhang, Aili Xing, Jingya He, Feng Wang, Zhongrui Li, Bin Sun

**Affiliations:** Department of Oral and Maxillofacial Surgery, School and Hospital of Stomatology, Jilin University, Qinghua Road 1500, Changchun, Jilin Province 130021 P. R. China

**Keywords:** Ameloblastoma, Conventional ameloblastoma, Conservative treatment, Traditional treatment, Curettage

## Abstract

**Background:**

Patients with mandibular conventional ameloblastoma undergoing radical surgical treatment experience greater trauma and often find it challenging to accept, whereas conservative therapy is associated with a higher recurrence rate. In this study, we have improved traditional conservative treatment for mandibular conventional ameloblastoma by curettage combined with bone cavity opening (Cur/BCO). This retrospective study aimed to evaluate the effectiveness of the Cur/BCO treatment by comparing its recurrence rate and bone mineral density (BMD) growth rate with the traditional conservative treatment approach.

**Methods:**

A total of 40 patients, meeting the study’s inclusion and exclusion criteria from 2012 to 2020, were screened, with 20 in the modified group and 20 in the traditional group. ImageJ (RRID: SCR_003070) software was employed for measuring image indices. All data were analyzed using T-test, Chi-square test and Fisher exact test in SPSS 26.0 (*p* = 0.05).

**Results:**

The incidence of recurrence was significantly lower in the modified group, at only 5%, compared to 35% in the traditional group (*p* < 0.05). Regarding bone mineral density (BMD) growth rate, the average value in the modified group was 0.0862 ± 0.2302 (/month), significantly higher than the average value of 0.0608 ± 0.2474 (/month) in the traditional group (*p* < 0.05).

**Conclusions:**

In this study, it was found that the recurrence rate of the modified conservative treatment (Cur/BCO) was lower than that of the traditional conservative treatment for managing mandibular conventional ameloblastoma. Furthermore, the BMD growth rate was quicker in the modified group. Thus, Cur/BCO could be considered as a viable option for the conservative treatment of mandibular conventional ameloblastoma.

## Background

Ameloblastoma is a common odontogenic tumor of the jaws that can arise from the enamel organ, remnants of the dental lamina, the lining of an odontogenic cyst, or perhaps the basal epithelial cells of the oral mucosa [1, 2]. It typically manifests as a slow-growing, painless swelling that may lead to cortical bone bulging, malocclusion, teeth loosening, and potentially severe facial deformities [3]. The World Health Organization (WHO) revised its classification in 2017, dividing it into three sub-types: conventional ameloblastoma, unicystic, and extraosseous/peripheral types. It redefined the former solid and cystic ameloblastoma as conventional ameloblastoma. The 2022 edition is based on the fourth edition with the addition of adenoid ameloblastoma [4, 5]. The most prevalent type with a potentially considerable recurrence risk is conventional ameloblastoma, a benign yet locally invasive tumor primarily affecting the body and posterior region of the mandible in patients within their third and fifth decades of life [6–8].

The surgical treatment remains the most efficacious approach to managing mandibular conventional ameloblastoma, but whether to use a radical or conservative approach is still debatable. Radical surgery primarily involves bone resection, necessitating a segmental resection with a minimum 1.0 cm margin to the bone, including a soft tissue border [9]. Although the recurrence rate of radical surgery is low, around 8% [10], it is extremely traumatic to patients, particularly for young people, leading to severe facial deformities and functional sequelae that necessitate simultaneous reconstruction using autologous bone grafts or other materials [11, 12]. Given that conventional ameloblastoma is principally benign and rarely life-threatening, radical surgery might be considered as “overtreatment” and prioritizing recurrence reduction at the expense of patients’ quality of life does not constitute a reasonable strategic approach. In recent years, an increasing number of surgeons have preferred conservative surgical approaches for the treatment of conventional ameloblastoma, complemented by regular and stringent evaluation and long-term follow-up [13]. Conservative surgery includes enucleation, curettage, and marsupialization individually or in combination with enucleation or curettage, as well as enucleation or curettage plus other adjuvant therapies, such as decompression, cryotherapy, or Carnoy’s solution [14]. These traditional conservative approaches can effectively preserve the integrity of the mandible and avoid deformities and dysfunction effectively [15]. However, they possess a high recurrence rate, reaching up to 41% [10, 16]. Consequently, it is imperative to identify a surgical method that minimizes trauma and recurrence while also taking into account the patient’s quality of life.

**Fig. 1:**
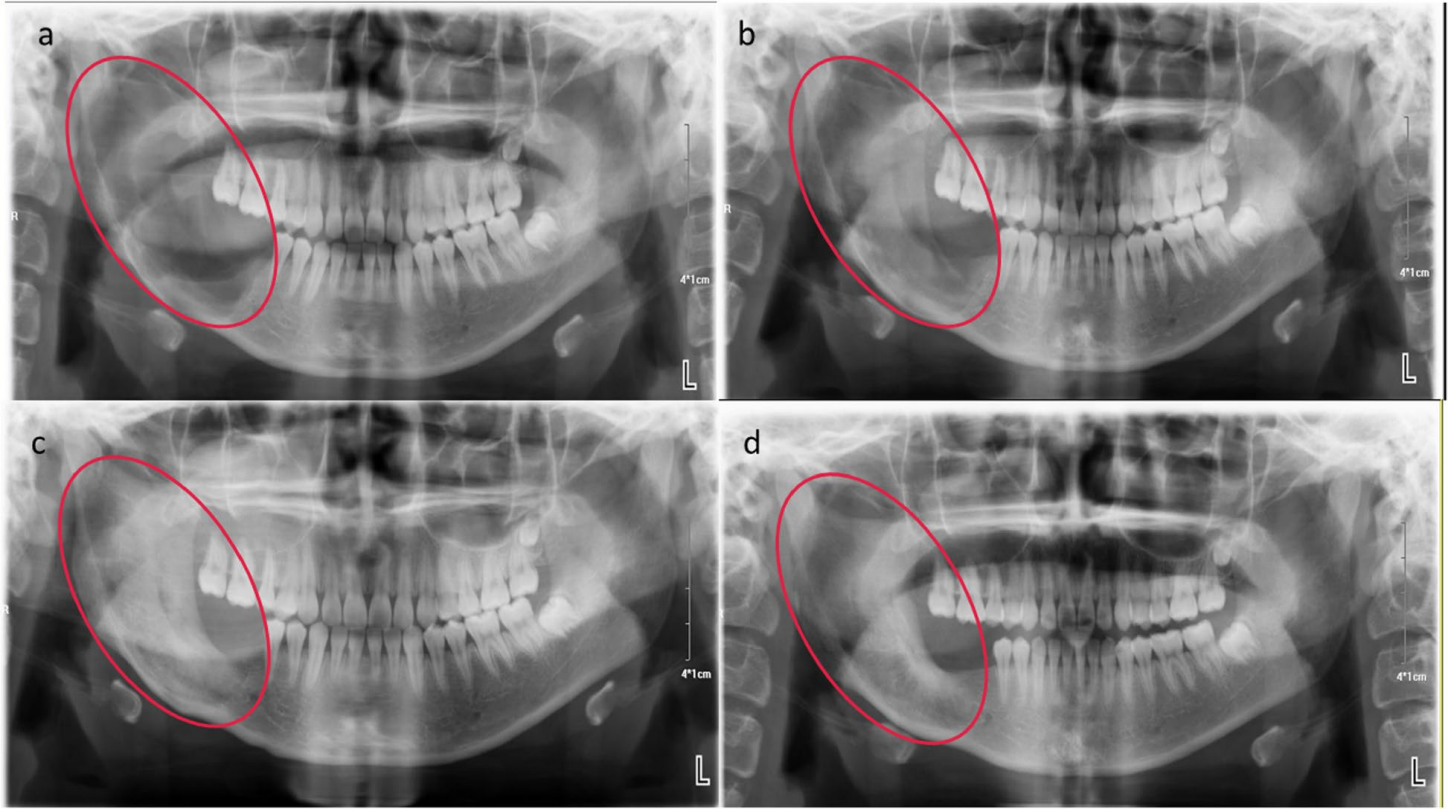
Preoperative and postoperative comparative panoramic image of patient undergoing the curettage combined with bone cavity opening. Preoperative panorama (**a**), postoperative # 3 months (**b**), postoperative # 6 months (**c**), postoperative # 1 year (**d**)

For more than five years, our hospital has applied a modified conservative method, curettage combined with bone cavity opening (Cur/BCO), for the treatment of mandibular conventional ameloblastoma, yielding satisfactory outcomes (Fig. 1). In the present study, we conducted a retrospective comparative analysis of the clinical data from two methods (Cur/BCO VS Curettage combined with iodine tincture cautery) for managing mandibular conventional ameloblastoma, focusing on the recurrence rate and growth rate of bone mineral density (BMD) to objectively evaluate the efficacy of this modified surgical technique.

## Methods

### Research design and data collection

This study followed the 1964 Declaration of Helsinki and its later amendments on medical protocol and ethics, and the regional ethical review board of the School and Hospital of Stomatology, Jilin University approved the study. All the patients received systematic treatment in the Department of Oral and Maxillofacial Surgery of the hospital.

All patients diagnosed with mandibular conventional ameloblastoma by postoperative pathology and image data in Stomatology Hospital of Jilin University from 2012–2020, all their clinical and image data were collected and reviewed, and telephone follow-up was conducted. Inclusion criteria: (1) the diagnosis was conventional ameloblastoma according to the WHO recommendation and occurred in the mandible, (2) the surgical methods were Cur/BCO or curettage combined with iodine tincture cautery, (3) no systemic or local diseases affecting bone metabolism, such as osteomyelitis, active moderate or severe periodontitis, etc. (4) follow-up for more than 24 months, and the patients voluntarily participated in the follow-up investigation by signing informed consent, (5) the clinical information and image data for the patients were complete. Exclusion criteria: (1) patients diagnosed with non-mandibular conventional ameloblastoma, (2) surgical options were not Cur/BCO or curettage combined with iodine tincture cautery, (3) patients with systemic diseases affecting bone metabolism or poor general condition, (4) patients were followed up for less than 24 months, and patients who did not wish to be followed up, (5) patients’ medical records were incomplete, follow-up was lost, etc.

**Fig. 2:**
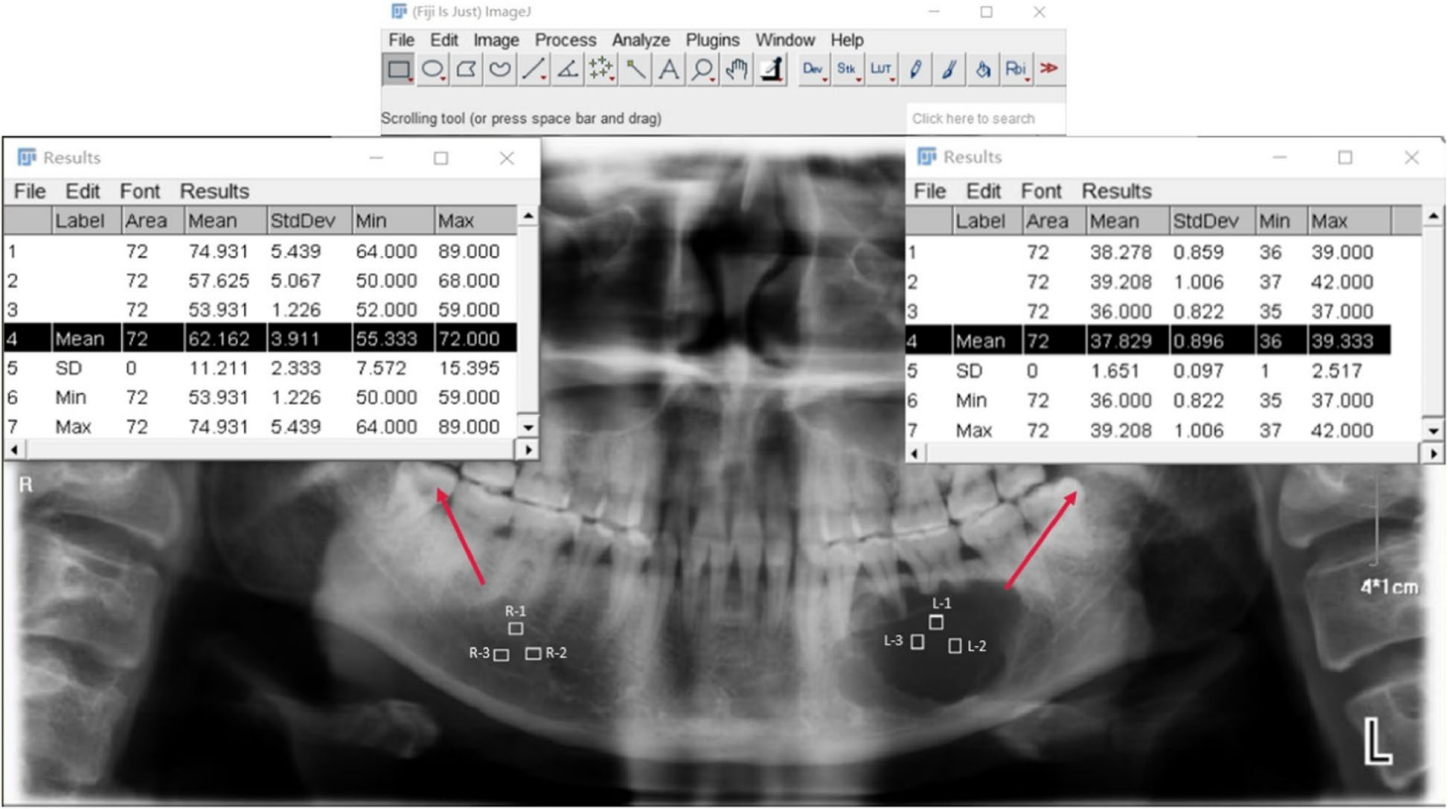
The gray values of L-1, L-2, and L-3 regions were selected from the panorama of the patient to calculate the average gray value of the lesion area. Similarly, the gray values of R-1, R-2, and R-3 regions represent the average gray value of the healthy bone in the symmetric region

All image data were taken by professionals in the Department of Radiology, Hospital of Stomatology, Jilin University. The patients were all photographed in a standardized position using the same machine, under the guidance of experienced medical professionals. Exposure conditions and doses were the same for all subjects. The images of all subjects were saved in JPG. format and collected for this retrospective study. In this study, we quantified the gray value of pre-surgery and 6-month post-surgery images for each patient to determine the rate of BMD growth, resulting in a more accurate depiction of BMD growth. The gray values of all images were measured using Image J software, and the average gray value in each lesion area and the symmetric normal area was measured. The ratio between the two was defined as the corrected gray value. The correction value can mitigate the confounding effects of variables such as mandible position, age, and sex, thereby enhancing the accuracy of the data. The lesion was divided into three distinct regions based on their midpoints, and each region’s midpoint served as the designated measurement point. These points were consistent in image measurements taken both before and after surgery. The measurements were replicated three times, with the average of the three measurements being defined as the mean corrected gray value. The difference between the mean corrected gray value post-operation and the mean corrected gray value before operation was utilized to represent the change in BMD growth, and the increased rate of BMD growth was the change in BMD growth divided by time. The measurement time was recorded in months, and images taken 6 months after the operation were selected for evaluate (Fig. 2).

### Surgical procedure and management

#### Modified conservative treatment: curettage combined with bone cavity opening (Cur/BCO)

**Fig. 3:**
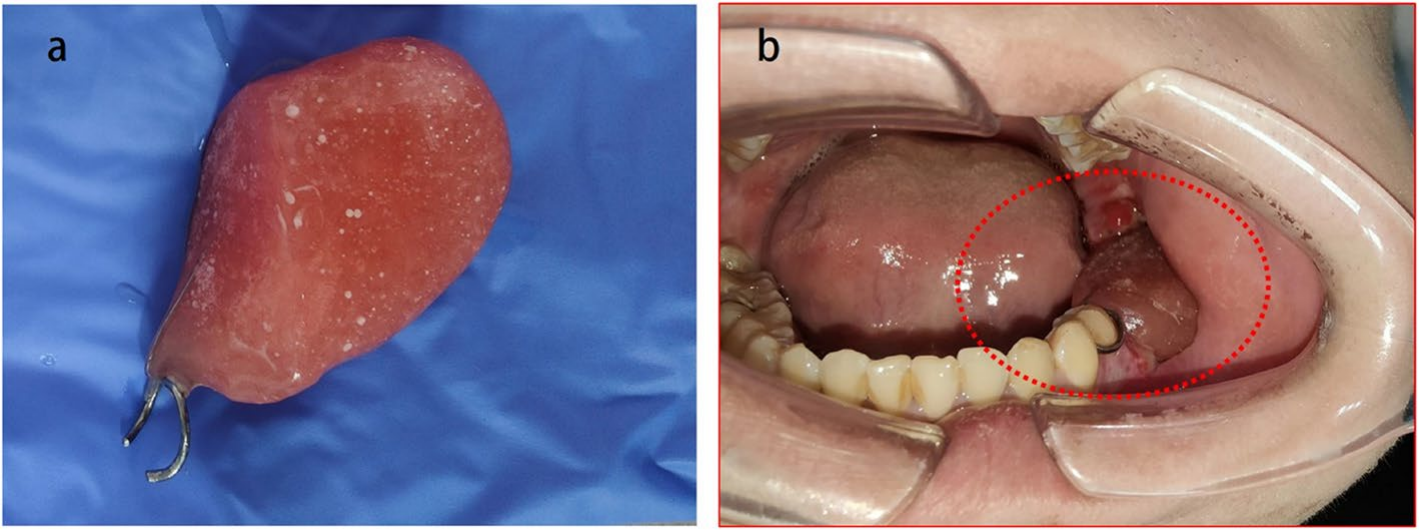
The self-condensing plastic plug (**a**), within the bone cavity 4 -5 days after surgery (**b**)

Surgeries were conducted under general anesthesia via an intraoral approach. The first curettage procedure involved thoroughly cleaning the lesion without employing any adjuvant therapies. Teeth extraction was performed when necessary. The second step entailed the removal of the mucosa and soft tissue above the cavity, creating a window that corresponded to the size of the tumor to preclude the cavity from closing and altering the previously established hypoxic environment. Titanium plates were used if pathologic fractures occurred during the operation. Subsequently, the bone cavity was filled with iodoform gauze. The iodoform gauzes were removed on postoperative days #4–5 and replaced with a self-condensing plastic plug to prevent the surrounding soft tissue from shutting the bone cavity window (Fig. 3). The patients were instructed to irrigate the bone cavity two or three times daily with 0.9% (20 ml) normal saline solution.

#### Traditional conservative treatment: curettage combined with iodine tincture cautery

The operations were likewise conducted under general anesthesia. The first step is similar to the modified surgical approach. Subsequent to curettage, the bone cavity was meticulously polished using a drill, followed by the application of a 3.0% iodine tincture solution for intraluminal cauterization. Suturing the mucosa to cover the cavity was the final procedure. Sometimes, iodoform gauze was employed for drainage, which was removed on postoperative day #3, allowing the wound cavity to naturally close thereafter.

### Statistical analysis

The continuous variables were expressed as the means ± standard deviations (SD) and percentages were used to express categorical variables. Comparisons between subgroups were performed using the T-test, Chi-square test and Fisher exact test. Statistical analyses were performed by SPSS 26.0 for Windows. A *P*-value less than 0.05 was considered to be statistically significant.

## Results

**Fig. 4:**
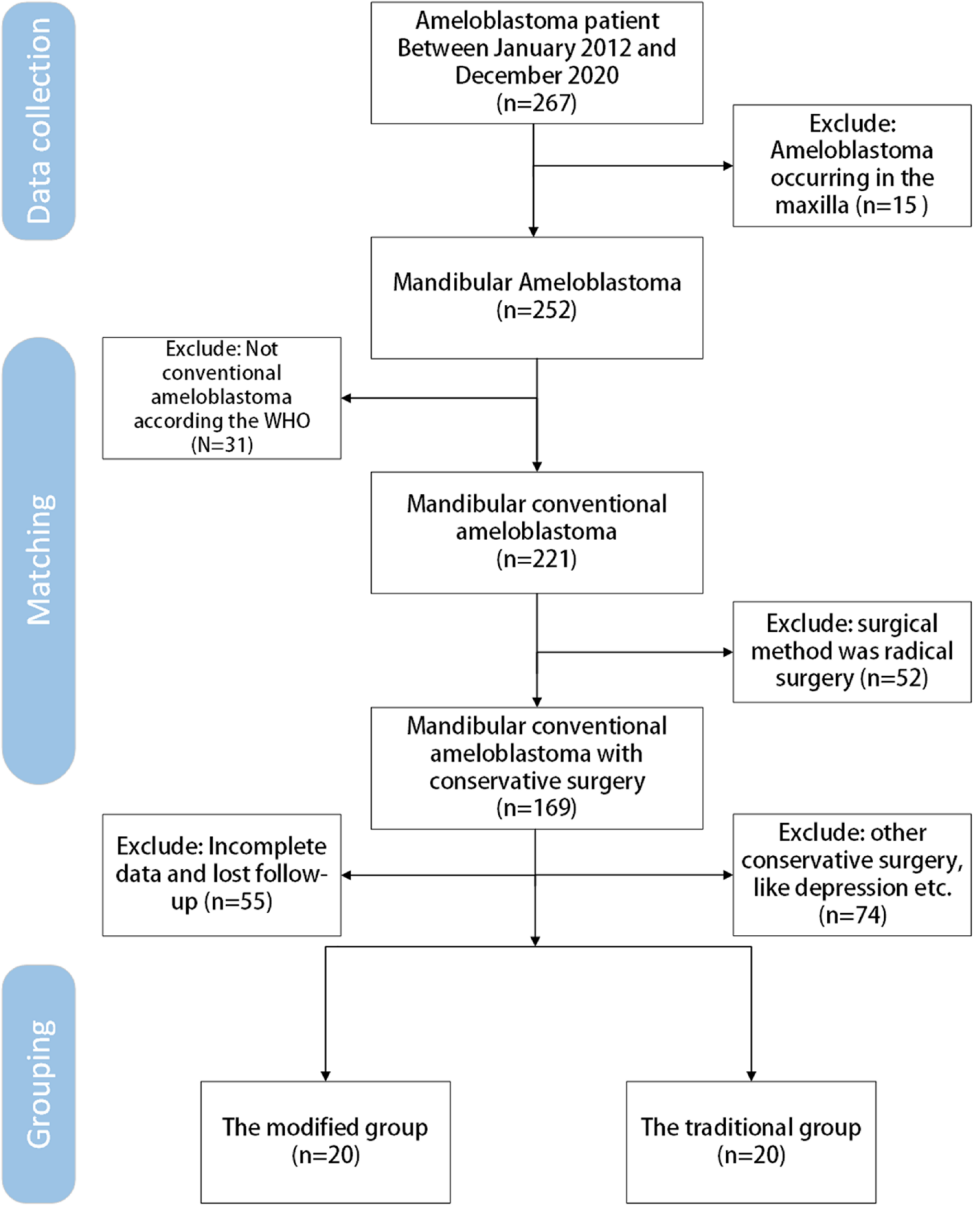
Flowchart

In the present study, a total of 40 subjects satisfying the inclusion and exclusion criteria were screened. Of these, 20 subjects in the modified group received Cur/BCO treatment, while 20 subjects in the traditional group underwent curettage combined with iodine tincture cautery. The flowchart of screening patients is shown in Fig. 4.

Of the 40 patients, 21 were male and 19 were female. The average age of the modified group was 27 y ± 10.04, and that of the traditional group was 33 y ± 11.09. Tumor lesions in all patients were predominantly located in the mandibular molar area, basically centered on third molars, and frequently involved the mandibular ramus or body. The average tumor area of the modified group was 15.94 cm^2^ ± 10.10, and that of the traditional group was 11.04 cm^2^ ± 6.89. The average tumor size of all patients was 13.49 cm^2^ ± 8.88. For the remaining clinical manifestations, both groups exhibited lower lip numbness, with a low prevalence of 5% in the modified group and 10% in the traditional group. Pathological fractures were absent in both groups. The continuity of the mandible was effectively maintained following both surgical interventions. The patients were monitored for a period of 36 to 77 months, with an average follow-up duration of 52 months ± 11.67 in the modified group and 49 months ± 7.18 in the traditional group. There was no significant difference in the basic clinical information between the above two groups (Table 1).

**Table 1:** Basic clinical information of all the patients

	Modified group (*N* = 20)	Traditional group (*N* = 20)	*P*-value
Gender			0.752
Male	10 (0.5)	11 (0.55)	
Female	10 (0.5)	09 (0.45)	
Age	27 ± 10.04	33 ± 11.09	0.058
Tumor site			0.723
Ramus	5 (0.25)	6 (0.33)	
Molar	15 (0.75)	14 (0.67)	
Tumor size	15.94 ± 10.10	11.04 ± 6.89	0.081
Clinical symptoms			
Lip numbness	1 (0.05)	2 (0.10)	0.256
Pathological fracture	0 (0)	0 (0)	
Follow-up time	52 ± 11.67	49 ± 7.18	0.419

Data are shown as the mean ± standard deviation or as the number (percentage)

**Fig. 5:**
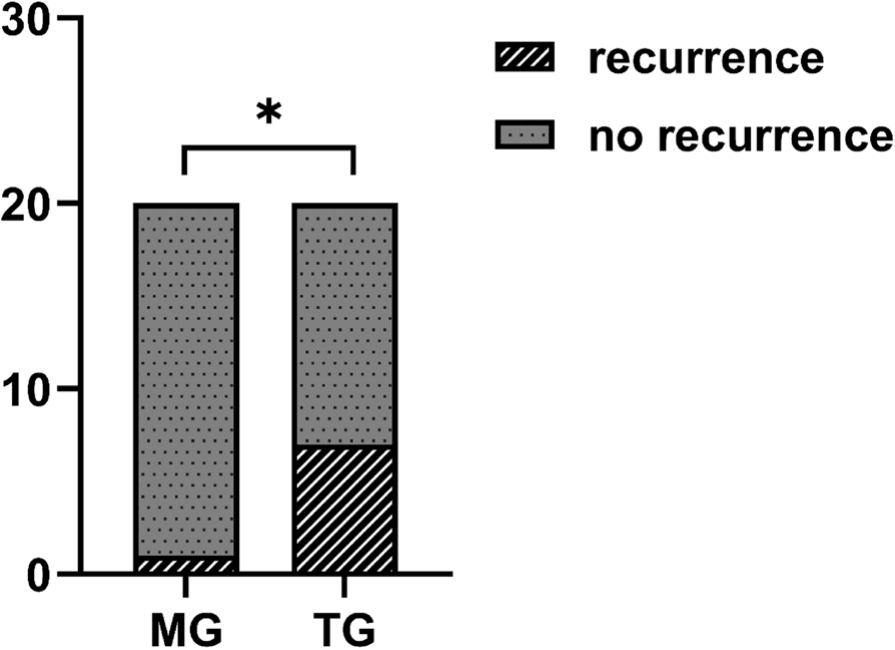
One patient in the modified group (*n* = 20) recurred, compared with seven in the traditional group (*n* = 20). MG, modified group; TG, traditional group. *Statistical significance at *p* < 0.05

The recurrence rate of the two groups demonstrated a statistically significant difference, with only 1 patient (5%) in the modified group experiencing a recurrence, in contrast to 7 patients (35%) in the traditional group (Fig. 5). The recurrence of patients in the modified group occurred 8 months post-operation, potentially attributed to inadequate adherence to plug usage and unscheduled wound irrigation, leading to premature healing of the wound cavity. This led to the formation of an anoxic environment within the wound cavity, promoting the active growth of residual tumor cells, and ultimately contributing to the recurrence. The patient underwent a second modified surgical intervention, and no recurrence has been observed until now. The gray value was measured using Image J software, and the BMD growth rate of the two groups of patients was calculated according to the established formula. The BMD growth rate in the modified group was 0.0862 ± 0.2302 (/ month), while that in the traditional group was 0.0608 ± 0.2474 (/ month). A statistical difference was observed between the two groups (Table 2).

**Table 2:** Recurrence rate and BMD growth rate in the modified group and traditional group

	Modified group (*N* = 20)	Traditional group (*N* = 20)	*P*-value
Recurrence	1 (0.05)	7 (0.35)	
No recurrence	19 (0.95)	13 (0.65)	
Total	20	20	0.018^*^
BMD growth rate (the mean corrected gray value/month)	0.0862 ± 0.2302	0.0608 ± 0.2474	0.002^*^

Data are shown as the mean ± standard deviation or as the number (percentage)

*BMD* bone mineral density

^*^Statistical significance at *p* < 0.05

## Discussion

Ameloblastoma predominantly occurs in young people [2, 17]. From the results, the average age of the two groups was less than 35 years. There is no predilection for males or females [6]. In this study, 21 patients were male, and 19 patients were female. The mandible, particularly the molar area and the angle, is the most commonly affected site [18]. All patients exhibited involvement in the mandibular molars and ramus. The mean tumor size was approximately 13.49 cm^2^ ± 8.88. Neither group reported pathological fractures, nor were titanium plates used for mandibular support. Numbness in the lower lip was prevalent in both groups, potentially attributed to the traction of the mandibular nerve during the operation. This, however, was also influenced by the size and location of the tumor. It has been reported that the mean time for ameloblastoma recurrence is 34 months after surgery [19]. In our study, the follow-up period ranged from 36 to 77 months. Our minimum follow-up time surpasses the mean recurrence time, allowing for a comprehensive and effective assessment of the emergence of recurrent cases. In our study, almost all the imaging findings presented a multi-chamber pattern, without honeycomb or locally malignant types. Based on the aforementioned fundamental characteristics and the surgeon’s extensive clinical experience, the authors propose that conservative treatment may be indicated under certain conditions. Regarding imaging, the lesion should exhibit neither honeycomb nor local malignancy features, and nearly all diseased tissue can be effectively removed during the surgical procedure. The extent of exposure to the open bone cavity depends on the size of the primary tumor. During the surgical procedure, all tumor tissues within each compartment should be completely excised. The intraoral wound should be designed with an appropriate size to preclude self-sealing of the wound. Although there is no specific tumor size threshold, the residual bone must be capable of supporting the mandible without inducing pathological fracture. Cur/BCO also present some potential postoperative complications. The first potential complication is infection. To mitigate this risk, the wound is filled with iodoform gauze 4–5 days post-surgery, which serves an anti-inflammatory and anti-corrosive function. Patients must consume liquid food, regularly clean their mouths, and maintain oral hygiene. To date, we have not observed any postoperative infections in our patients. The second potential complication is pathological fracture. Prior to surgery, we assess the patient’s residual bone and stringently control the indications to ensure that there is sufficient residual bone to support and prevent pathological fractures post-surgery.

**Fig. 6:**
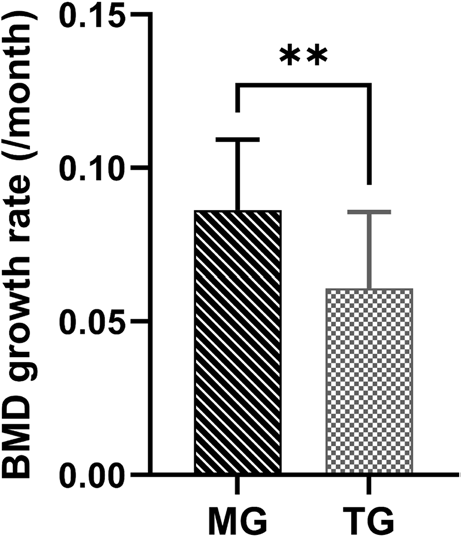
The BMD growth rate of the modified group and the traditional group was 0.0862 ± 0.2302 (/month) and 0.0608 ± 0.2474 (/month) at postoperation#6 months. BMD, bone mineral density; MG, modified group; TG, traditional group. *Statistical significance at *p* < 0.05

Patients were required to undergo regular panoramic imaging after surgery. Compared to computed tomography, panoramic imaging offers a more cost-effective and patient-friendly approach for effectively and intuitively monitoring bone regeneration and potential signs of recurrence, while minimizing radiation exposure for the patient. Upon confirmation of a pathologically diagnosed recurrence, prompt management in either a conservative or radical approach was implemented, depending on the specific case. In panorama images, a gray value could indicate BMD. As illustrated in Fig. 6, the comparison of BMD growth rate between the two groups demonstrated a statistically significant difference. Decompression, frequently employed to manage cysts and unicystic ameloblastoma, has been acknowledged as an efficacious conservative treatment, and bone regeneration occurs concurrently with decompression [20]. Ling Gao et al. employed a method analogous to assess the growth rate of BMD in unicystic ameloblastoma during decompression, which amounted to 9% [21]. Upon opening the window in the modified group, the BMD growth escalated by 0.0862/month, approximately reaching 8.62%. This is consistent with the findings of Ling Gao et al. Some adjuvant therapies, such as Carnoy’s solution and liquid nitrogen freezing of the cyst cavity, are frequently employed to eliminate residual infiltrative tumor islands of bone walls, as reported by Carneiro et al. However, their application might severely impair the regenerative capacity of bone tissue [22, 23]. As a strong fungicide, iodine tincture has the capacity to eradicate tumor cells and bone cells, thereby influencing bone development. This phenomenon might contribute to the explanation for the low BMD growth rate typically observed in traditional conservative treatments. The Cur/BCO technique is characterized by its approach of not closing the wound cavity and refraining from employing any adjuvant therapies during the operation.

In this study, the recurrence rate of patients in the traditional group was 35%, which was similar to previous research by Darshani Gunawardhana, K. S et al. [24]. In contrast, only 5% of patients in the modified group experienced a recurrence. As reported previously, the recurrence rate associated with radical treatment is approximately 8% [10]. Cur/BCO exhibits a recurrence rate that is practically as low as the radical treatment level, while causing less patient harm. Particularly in young patients, this approach minimizes detrimental impacts on facial development and psychological well-being [25]. Younger patients exhibit a higher propensity for conservative treatment. In comparison to radical surgery, this approach necessitates no additional fibula or iliac bone repair, and the subsequent bone height, width, and density are generally sufficient to meet the postoperative requirements of patients, such as for implantation. Although traditional conservative treatment presents the advantages of Cur/BCO, its recurrence rate is comparatively high, ranging from 18.2% to 48.7% [16, 26–28]. Precisely, conventional ameloblastoma is deemed as a notably recurrent and locally invasive odontogenic tumor [29]. It has been ascertained that the micro-environment assumes a crucial role in the growth or apoptosis of residual cells subsequent to tumor resection, and modifications in the tumor micro-environment can notably lessen the likelihood of tumor recurrence [30, 31]. Hypoxia is speculated to be the predominant state of the tumor microenvironment during its progression [32, 33]. Ameloblastoma is an odontogenic tumor in which oxygen is difficult to diffuse to all areas of the tumor when the volume of the tumor surpasses 1.0mm^3^ in the jaw bone [34]. The hypoxia area is scattered in the tumor tissue and is associated with tumor invasion and recurrence [35]. da Costa NM reported that special hypoxia regions existed in ameloblastoma, further emphasizing the relationship between tumor invasion and oxygenation [36]. In this study, Cur/BCO was employed in the modified group for the treatment of mandibular conventional ameloblastoma. Curettage was utilized to clear the tumor, followed by the removal of the surrounding mucoperiosteal to expose the bone cavity, allowing the air to fill the cavity connecting with the oral cavity until the lesion gradually contracted and healed. Most critically, this approach differs from the conventional conservative method of suturing the mucosal membrane to close the cavity. We hypothesize that the presence of abundant air in the bone cavity alters the micro-environment of residual tumor cells and the osteogenesis environment. This may account for the low recurrence rate of Cur/BCO. Further exploration of the specific mechanisms of Cur/BCO is necessary, and we will continue to pursue this in our future research.

The limitation of this study lies in the relatively small sample size due to the recent implementation and improvement of this surgical method (Cur/BCO) and it is not a prospective experiment. However, for ameloblastoma, there are certain difficulties in conducting prospective experiments.

## Conclusion

In this study, we comparatively analyzed two conservative treatment approaches. The findings indicate that Cur/BCO is a promising surgical approach for mandibular conventional ameloblastoma, characterized by a low recurrence rate and rapid BMD growth. This technique offers a novel therapeutic method and insights for the management of mandibular conventional ameloblastoma.

## Data Availability

Data and materials are available and accessible from the corresponding author on reasonable request.

## Abbreviations

Cur/BCO: Curettage combined with bone cavity opening
BMD: Bone mineral density
TG: Traditional group
MG: Modified group
